# Immunomodulatory and chemopreventive effects of resveratrol on the digestive system cancers

**DOI:** 10.32604/or.2024.049745

**Published:** 2024-08-23

**Authors:** MEIR DJALDETTI

**Affiliations:** Laboratory for Immunology and Hematology Research, Rabin Medical Center, Hasharon Hospital, Petah-Tiqva, the Sackler School of Medicine, Tel-Aviv University, Ramat Aviv, 69978, Israel

**Keywords:** Resveratrol (RSV), Chemoprevention, Digestive tract cancers, Immunity, Cell death, Polyphenols

## Abstract

Resveratrol (RSV), the primary polyphenol found in grapes, has been revealed to have anti-inflammatory properties by reducing the capacity of the peripheral blood mononuclear cells to produce pro-inflammatory cytokines, including IL-1β, IL-6, IL-1ra and TNFα. Considering the close association between chronic inflammation and cancer development, RSV’s immunomodulatory properties are one way by which the polyphenol may inhibit cancer initiation, proliferation, neovascularization, and migration. Resveratrol influences the generation of microtumor environment which is one of the key factors in cancer progress. In addition to immunomodulation, RSV inhibits cancer development by expressing anti-oxidant effects, causing cell cycle arrest, stimulating the function of certain enzymes, and activating cell signaling pathways. The end outcome is one of the various forms of cell death, including apoptosis, pyroptosis, necroptosis, and more, as it has been observed *in vitro*. RSV has been shown to act against cancer in practically every organ, while its effects on colon cancer have been documented more frequently. It is remarkable that longer-term clinical studies that may have established the potential for this natural substance to serve as a therapeutic adjuvant to traditional anti-cancer medications were not prompted by the encouraging outcomes seen with cancer cells treated with non-toxic doses of resveratrol. The current review aims to assess the recent findings about the immunological and anti-cancer characteristics of RSV, with a particular emphasis on cancers of the digestive tract, as a challenge for future clinical research that may contribute to the better prognosis of cancer.

## Introduction

The history of grape cultivation is estimated to be 6,000 to 8,000 old, based on archeological and seed findings [1]. The dual utilization of grapes as a delicious fruit and as a source for extensive vine production explains their widespread farming and development. Leaving apart the high commercial value of the grapes in the nutritional and vine industry, grapes consumption is associated with beneficial health virtues. Studies have shown that grapes feeding diminishes the risk of cardiovascular and skin diseases, diabetes, neurodegeneration, Alzheimer disease, obesity, and certain types of cancers [2–4]. These effects result from grapes’ high polyphenol content, of which RSV is the most significant. Plants that produced RSV were significant items in the Asian pharmacopeia, according to Venkat et al. [5].

RSV has demonstrated anti-inflammatory properties by mitigating the harmful effects of reactive oxygen species (ROS), primarily hydrogen peroxide and superoxide radicals [6]. For middle-aged and older people with increased oxidative and anti-inflammatory activity, RSV plays an important role. It was shown that treatment of their peripheral blood mononuclear cells (PBMC) with RSV regulated the production of the pro-inflammatory cytokines IL-6 and TNFα [7]. RSV inhibits the activity of immune recognition receptors (TLR), such as TLR4, which stimulates the inflammatory mediators nuclear factor kappa B (NF-kB) and interferon (IFN) [8]. Given the proven impact of chronic inflammation on the onset and progression of cancer [9,10] and the immune system’s active participation in this process, it is plausible that the immunomodulatory properties of polyphenols play a significant role in cancer prevention. Inflammatory reactions are heightened by malignant processes in general and digestive tract malignancies in particular. This, in turn, activates immune system components such as PBMC, initiating a cross-talk between immune and cancer cells [11].

A comprehensive review of RSV pharmacokinetics has been conducted by Neves et al. [12]. Given orally it is readily absorbed, however, each person’s absorption is different. In the digestive system, it is converted by the intestinal and liver cells into glucuronic acid and sulfate coupled with the phenol clusters and rapidly excreted through the feces and urine suggesting a low bioavailability. To avoid this impediment, efforts have been made to provide its consistent delivery using RSV-loaded nanoparticles which showed encouraging outcomes by inhibition of colon cancer cell migration and triggering apoptosis [13].

RSV may induce cancer cell death through a variety of mechanisms such as apoptosis, pyroptosis, ferroptosis, cuproptosis, necrosis, and more [14]. With an emphasis on digestive tract cancers, the current work aimed to review the immunomodulatory and carcinopreventive effects of RSV in connection to the beneficial effect of polyphenols on the prevention of tumor development.

## Resveratrol as an Immunomodulator

There is sound evidence that RSV exerts immuno-regulatory functions. RSV reduces the immune cell expression of pro-inflammatory cytokines by inhibiting of toll-like receptor (TLR) [15]. It regulates the immune properties of lymphocyte subtypes having a crucial role in the process of inflammation [16]. RSV induces polarization and immune response of CD8^+^T lymphocytes and the production of cancer preventing IFNγ and TNFα cytokines [17]. Acting on CD8^+^T cells by upregulating the levels of TNFα, IL-2, and IL-12, RSV enhanced their cytotoxic effect, an important step in halting the growth of cancer [17–20]. By upregulating protein kinase B (Akt B) and the mammalian rapamycin target (mTORC2) pathway, RSV enhanced the function of the natural killer (NK) cells that actively participate in the process of carcinoprevention [21].

After being treated with RSV, neutrophils, and macrophages exhibited a concentration-dependent reduction of ROS generation [22]. When unstimulated human PBMC were incubated with RSV, the production of IL-6, IL-1ra, and IL-10 was lowered, while TNFα release was slightly elevated [23]. Conversely, exposing unstimulated RAW-264.7 macrophages to RSV increased IL-10 release and decreased TNFα production [24], indicating that the polyphenol has distinct effects on various macrophage subtypes. When human THP-1 monocytes and macrophages were treated with RSV, a difference in the immune response was seen; although the polyphenol stimulated the pro-inflammatory cascade and inhibited monocyte proliferation by causing cell cycle arrest in the S phase, it also produced an anti-inflammatory burst in THP-1 macrophages [25].

The pro-inflammatory cytokines IL-6 and TNFα were significantly inhibited when lipopolysaccharide (LPS) stimulated macrophages were treated with RSV [26], whereas the generation of the anti-inflammatory cytokines IL-10 and TGF-β was enhanced [27]. Further explanations for the anti-inflammatory activity of RSV treated macrophages include inhibition of NF-kB, Janus kinase, and signal transducers and transcription activators (JAK/STAT), as well as inhibition of IL-6 and TNFα secretion by increased production of cytokine signaling suppressor 1 (SOCS1), being a STAT inhibitor [28,29]. These findings are consistent with the decreased generation of inflammatory mediators achieved with RSV, such as TNFα, IL-8, and monocyte chemoattractant protein-1 (MCP-1) through activation of NF-kB, JAK/STAT, and Janus kinase in LPS stimulated monocytes [30].

**Figure 1:**
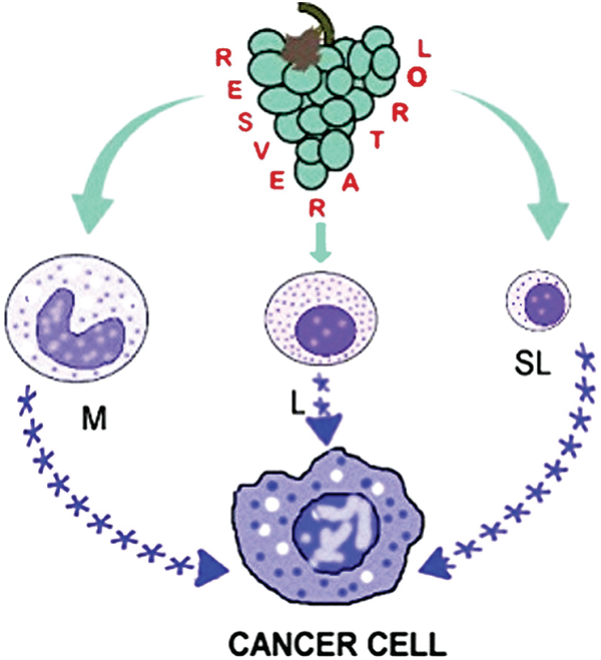
Peripheral blood mononuclear cells are stimulated by resveratrol to release anti-inflammatory cytokines, which subsequently prevent chronic inflammation and the growth of cancer cells. M-monocytes and macrophages, L-large lymphocytes, SL-small lymphocytes, and asterisks denote anti-inflammatory cytokines.

Inhibition of vascular endothelial growth factor suppressor (miR-146a), activation of nuclear relation factor 2 (Nrf2), and suppression of hypoxia-inducible factor 1α (HIF-1α) are additional mechanisms to account for the anti-inflammatory activity of LPS stimulated RAW 264.7 macrophages exposed to RSV [31,32]. RSV affects M1/M2 macrophage polarization resulting in the inhibition of pro-inflammatory cytokine production and amplification of the anti-inflammatory ones [17,33,34] in part via adenosine monophosphate-activated protein kinase (AMPK) activation [35]. RSV suppresses the function of the pro-inflammatory M1 macrophages by activation of poly (ADP-ribose) polymerase (PARP), an effect amplified by the addition of nicotinamide [36]. These and other investigations provide strong evidence for RSV’s immunomodulatory function as a carcinopreventer. Fig. 1 illustrates the way RSV induces PBMC to produce anti-inflammatory cytokines that affect cancer cells and the tumor microenvironment.

## Resveratrol as Cancer Suppressor

A considerable number of studies suggest that RSV functions as a suppressor of cancer cell proliferation and has the potential to prevent their spreading to practically all organs [37,38]. Regrettably, while most of the studies have been carried out *in vitro* or with animal models using various types of cancer cells, clinical research has been conducted mostly on patients with colorectal cancer [39]. In addition to immunomodulatory activity, RSV prevents cancer development by affecting tumor microenvironment and inactivating essential pathways such as NF-kB, the mitogen-activated protein kinase (MAPK), and JAK/STAT that are essential for cancer cells’ development [40–42]. Inhibition of ROS species and pSTAT-3 expression in the gut microbiota by RSV play an important role in carcinoprevention [43].

Cancer development is closely associated with the function of tumor-associated macrophages (TAM). It has been reported that the M2 TAM polarization was affected by RSV due to a significant decrease in STAT3 activity, an effect observed both in lung cancer cells and in a mouse lung cancer model [44]. Promoting M1 to M2 macrophage polarization, lesser production of IL-6, and suppression of STAT3 pathway by RSV was observed in breast cancer cells [45]. Enhanced chemosensitivity, as well as inhibited differentiation, migration, and vascular endothelial growth factor (VEGF) production, were also the result of activated M2 polarization when tumor cells were incubated with RSV, or with the conditioned medium obtained after incubation of M2 macrophages treated with the polyphenol [46]. Additional factors such as angiogenesis, epithelial-mesenchymal transition, and repressing the functions of cancer stem cells targeted by RSV contribute to preventing cancer development and metastasis [47].

**Figure 2:**
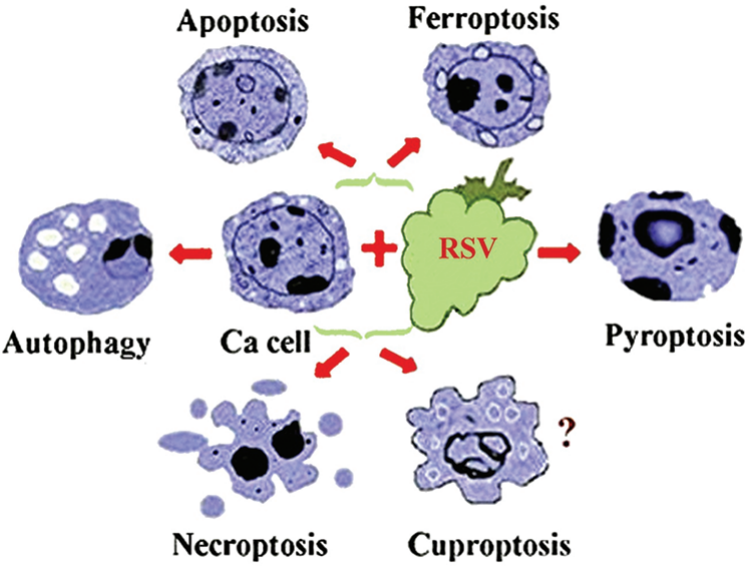
A schematic presentation of some of the mechanisms by which resveratrol may induce cancer cells’ death. The knowledge about the impact of RSV on cuproptosis is limited, as shown by the question mark. Ca cell-cancer cell.

Overcoming cancer cells’ radioresistance is another benefit granting RSV the role as an adjuvant to radiotherapy [48]. Moreover, it has been demonstrated that RSV increases the susceptibility of breast cancer cells to talazoparib by simultaneously inhibiting Akt signaling and increasing autophagy [49]. RSV may induce programed cancer cells’ death in 11 different ways [50], the most explored being autophagy, apoptosis, ferroptosis, pyroptosis, cuproptosis, and necroptosis [51] schematically presented in Fig. 2.

***Autophagy*** is a process by which RSV may inhibit cancer cell proliferation and metastasis [52], acting through a number of autophagic signaling pathways [53]. Sirtuin 3 (SIRT3), the protein that maintains mitochondrial homeostasis plays an important role in the regulation of autophagy, a process promoted by RSV in 4T1 breast cancer cells by upregulation of the SIRT3/AMPK autophagy pathway [54]. However, in certain types of cancer, SIRT3 may function as a tumor promoter [55]. Activation of the tumor suppressor nerve growth factor receptor (NGFR-AMPK-mTOR) pathway by RSV promoted both autophagy and apoptosis in A549 non-small lung cancer cells [56]. Restraint of p62 autophagy suppressor and mTOR activation is an additional mechanism by which RSV inhibits cancer progression [57,58]. Autophagy in human leukemia HL-60 cells induced by RSV was found to be dependent on a serine-threonine kinase (LKB1)-AMK and phosphatidylinositol-3-kinase (PI3K)/AKT-regulated mTOR pathways [59].

***Apoptosis*** plays a key role in cancer cells’ death. Depending on the cancer cell type RSV may induce apoptosis triggering a chain reaction by activating AMPK. Consequently, it promotes enhancement of the tumor suppressor TSC2 that inhibits the anti-apoptotic activity of mTOR. Stimulation of apoptotic receptors such as ROS and activation of P53 are additional mechanisms by which RSV sets off the procession of apoptosis [60]. Treating HCT-116WT colorectal cancer cells with RSV at doses >10 µM induced apoptosis by inhibition of the tumor promoter SIRT-1, increasing the expression of P21, P53, and the Bcl-2-associated x protein (Bax-2) [61]. Increased caspase activity and DNA breakdown induced by RSV lead to apoptosis expressed by condensation of nuclear chromatin and nuclear damage [62]. Tumor long non-coding RNAs linked with cell proliferation, spreading, and metastasis are also targeted by RSV [63]. Applied in combination with anti-cancer drugs, RSV increases cancer cells’ chemosensitivity by boosting apoptosis [64].

***Ferroptosis***, another mode of programmed cell death may be triggered by natural polyphenols including RSV [65]. The primary mechanism leading to ferroptosis is intracellular iron accumulation causing cell damage and death. The main ferroptosis regulators are amino acid transporters that exchange cysteine uptake for glutamate (SLC7A11), glutathione peroxidase 4 (GPX4), p53, NRF2, the heat shock proteins HSPB1, CISD1, the protein-coding FANCD2 and the converter of long-chain fatty acids and coenzyme A into fatty-acid coenzymes (ACSL4) [66,67]. RSV has the potential to cause ferroptosis in colorectal cancer cells through upregulating the formation of glutathione reductase 4 (GPX4), decreasing the expression of the cystine transporter solute carrier family 7 member 11 (SLC7A11), and increasing ROS production and lipid peroxidation [68]. Notably, RSV can enhance ferroptosis by acting as a SIRT inducer [69]. Therefore, activation of ferroptosis by RSV may serve as an additional adjuvant for the therapy of many diseases including cancer prevention.

***Pyroptosis*** is an alternative mechanism of cell death that is seen in various illnesses, including cancer. The role of pyroptosis in cancer cell death initiated extensive research. The process impacts the proliferation, invasion, and metastasis of cancer cells and is triggered by inflammasomes, gasdermin D cleavage, and elevated IL-18 and IL-1β cytokine production [62]. In a recent study, Huang et al. [70] brought attention to the capacity of dietary polyphenols, including RSV, to induce pyroptosis in cancer cells and in those in the tumor microenvironment via different pathways contingent on cancer type. RSV was able to induce pyroptosis by suppressing the activation of the NLRP3 inflammasomes bearing a sensor protein of the NLR family which is one of the key regulators of the inflammatory and innate immune response [71]. Pyroptosis could be enhanced in HT-29 and HCT116 colon cancer cells by certain drugs such as lobaplatin [72].

***Cuproptosis*** is a recently described mechanism that may overcome cancer cells’ drug resistance and leads to cell death by transfer of cupper ionophores into the cells causing cupper accumulation [14] and dysregulation of mitochondrial respiration. Copper overload induces membrane damage, mitochondrial vacuolization, chromatin condensation, and finally cell death [73]. The effect of cuproptosis is closely associated with the activity of the ferrodoxin 1 (FDX1) gene which is downregulated in several types of cancers. Low FDX1 expression correlates with poor prognosis [74]. Copper ions activate the PI3K pathway which in turn suppresses Akt activation [75]. According to Wang et al. [76], cuproptosis dependent on a number of copper-related genes is one of the causes of lung carcinoma cell viability, their detection being of prognostic value. Interestingly, copper-induced cell death caused by curcumin has been reported in HCC cells [77], but elucidating the effect of RSV and other polyphenols on cuproptosis in cancer cells, in general, presents a challenge.

***Necroptosis*.** Galluzi et al. [78] defined necroptosis as a type of programmed cell death dependent on serine-threonine kinase 3 (RIPK3) and mixed lineage kinase domain-like (MLKL). The authors highlighted the molecular mechanisms governing the process in a variety of diseases including malignancies. Because research suggests that necroptosis can function as both a tumor promoter and an inhibitor, its exact role in tumorigenesis is not fully clarified [79]. According to Lee et al. [80] RSV reduced the viability of LNCap prostatic cancer cells by activation of both necroptosis and apoptosis by raising the receptor interacting protein 3 (RIPK3) and mixed-lineage kinase domain like (p-MLKL) levels. This effect was more pronounced when the cells were treated with both RSV and docetaxel.

Future studies must clarify the mechanisms by which RSV causes cancer cell death and the dosage required to accomplish this objective in humans.

## Resveratrol and Cancers of the Digestive System

### Esophageal cancer

Prior research on 594 patients with different types of esophageal cancer who were given a diet high in polyphenols demonstrated that quercetin and RSV had a dose-dependent protective effect against the growth of the malignancy [81]. RSV, at dosages of 10 mM/L for 24–96 h significantly slowed the development of esophageal cancer EC-9706 cells and caused apoptosis, these effects being dose- and time-dependent. Additionally, in rats receiving intraperitoneal injections of RSV for surgically produced esophagitis, there was a decrease in the progression of Barrett’s metaplasia to esophageal cancer following RSV administration compared to controls [82]. RSV caused apoptosis and enhanced ROS generation in three esophageal cancer cell lines, i.e., OE33, OE19, and FLO-1, whereas the B-cell lymphoma-2 (Bcl-2) apoptosis regulating protein levels were lowered [83]. Further investigation into the potential protective effects of RSV against esophageal cancer appears crucial, given the higher morbidity and death rates associated with the disease and the limited number of therapeutic options now available.

### Gastric cancer

Although primarily *in vitro*, the RSV immunomodulatory, anti-inflammatory, and anti-cancer characteristics have been explored in gastric carcinoma. Wang et al. [84] have provided a thorough account of the mechanisms by which RSV may limit the genesis, progression, and metastasis of gastric cancer. According to Yang et al. [85], RSV may block IL-6-induced oncogenic uncontrolled cell proliferation and apoptosis by restricting Raf-MAPK pathway activation, which in turn suppresses the growth of the SGC7901 gastric cancer cell line. Suppressing the Wnt signaling pathway had a similar result in MGC-803 gastric cells [86]. When applied to human gastric cancer SGC-7901 cells, RSV elevated the expression of Bax and Bcl-2 apoptosis regulators, downregulated PARP, activated caspase-3, and caused apoptosis. Furthermore, the *in vivo* proliferation of cancer cells was suppressed in the SGC-7901 xerograph [87]. In the same kind of cells, RSV exerted a dose-dependent reduction in cell viability and an increase in apoptosis because of elevated cleavage of caspases 3 and 8, increased apoptotic Bax, and decreased NF-kB [88]. The ability of RSV to induce apoptosis in gastric cancer cells is explained by down-regulation of the NF-kB signaling pathway, which is known to support cancer progression, suppression of the anti-apoptotic protein, and activation of the apoptotic caspases 3 and 8. Moreover, the polyphenol’s capacity to damage mitochondrial membranes intensifies this action [89]. As a result of decreased NF-kB and heparanase activity by resveratrol, AGS and MKN45 gastric cancer cells expressed lesser invasive function [90]. RSV inhibits gastric cancer cells’ proliferation, invasion and metastasis by targeting miR-155-5p that acts as a pro-inflammatory oncogene [91] and by inhibition of their TGF-β1 induced epithelial-mesenchymal transition potential [92]. When coupled with cisplatin RSV had a synergistic effect in inducing gastric cancer cells’ apoptosis and inhibition of cells metastatic capacity through elevated ROS levels and inhibited mitotic cycle at GO/G1 phase [93].

### Colorectal cancer

Colorectal cancer (CRC) is well-known for its high prevalence and mortality rate and its resistance to chemotherapy [88] Research has shown that adding polyphenols, such as RSV, to traditional therapeutic medications may be rather beneficial [94]. The effect of the conventional drugs on tumor size, cancer cells’ cytotoxicity, and apoptosis was discovered to be larger when RSV was admitted concurrently with chemotherapy, according to a recent study based on 21 appropriate publications dealing with animal models with colorectal cancer [95]. Both in CRC human histologic sections and human CRC HCT116 cell line, RSV reduced cell growth by AKT1 and IL-6 downregulation [96]. RSV suppresses the Wnt signaling pathway, as well as cyclins D1 and D2 which are closely related to the development of CRC cells [97], and activates the metastasis-suppressor Raf-1 (RKP) protein [98]. Similarly, repressing and Wnt/β-catenin activation RSV inhibits CRC cells’ proliferation and differentiation while inducing apoptosis [99]. By increasing ROS and Bax expression levels, and inducing Bcl-2 downregulation, RSV promotes the apoptotic pathway and reduces CRC cell viability [100]. Activation of AMPK by RSV reduces the epithelial sodium channels in HCT116 and HT29 human colon cancer cells with a subsequent decrease in their proliferation, migration, and invasion [101]. Additional molecular ways by which RSV may attenuate CRC development include SIRT1, P53, P21, BMP7, COX-2, NF-kB, epithelial-mesenchymal transition (EMT), and certain caspases [102]. Moreover, treatment of CRC cells with RSV suppressed their cross-talk with tumor microenvironment (TME) cells through activation of the Sirt1 pathway, thus abating cell survival and migration [103].

### Pancreatic cancer

Pancreatic cancer is a fatal illness due to challenges with diagnosis and drug resistance [104]. Efforts to prevent its development and migration by conventional chemotherapy are rather disappointing. Consequently, the chemopreventive properties of RSV in this instance would be advantageous. RSV seems to have the potential to slow the spread of pancreatic cancer in a number of ways. *In vitro* and animal studies showed that blocking NF-kB activation therapy with RSV decreased the development of pancreatic intraepithelial metaplasia and acinar-to-ductal metaplasia [105]. Research has shown that RSV may affect the cell division and metastatic ability of pancreatic stem cells which are extremely resistant to the drugs now in use. Inhibition of the hypoxia-inducible factor-1α (HIF-1α) is another mechanism by which RSV slows the development and progression of pancreatic cancer cells [106]. Notably, the anti-proliferative effect of RSV depends on the cancer cell type. While all three pancreatic cancer cell lines, i.e., EPP85-181P, EPP85-181RNOV, and AsPC-1 exhibited inhibited proliferation after treatment with RSV, in the mitoxantrone-resistant EPP85-181RNOV cells this effect was observed to a much greater extent. However, the inhibitory effect of RSV on cell proliferation in all three lines proceeded through altered levels of the Bcl-2 pro- and anti-apoptotic proteins [107]. Qin et al. [108] reported a close relationship between autophagy and oxidative stress in relation to the nutrient-deprivation autophagy factor-1 (NAF-1) found in pancreatic cancer tissue and cancer stem cells. The authors revealed that inhibition of NAF-1 activity by RSV leads to inhibited cell migration and invasion. RSV applied concurrently with other phytochemicals to human pancreatic cancer MA-Pa-Ca-2 cells enhanced the activation of the cells’ resistance to oxidants regulator Nrf2 at a higher level than acting alone [109]. Remarkably, it was recently demonstrated that artificially produced RSV derivatives could inhibit the proliferation of pancreatic cells by causing DNA damage, apoptosis, and cell cycle arrest while not affecting normal pancreatic cells [110].

### Hepatocellular carcinoma

RSV is a useful adjuvant to the currently available medications for the treatment of hepatocellular carcinoma (HCC) because of its anti-inflammatory and anti-oxidative health qualities [111,112]. Considering the connection between inflammation and HCC development, one method of preventing the development of cancer is through RSV’s capacity to inhibit NF-kB activity, which is the catalyst for the start of the inflammatory cascade and the production of pro-inflammatory cytokines TNFα, IL-1α, IL-1β, and IL-6. Moreover, RSV increases the apoptosis of HCC cells by inhibiting the PI3/Akt/mTOR pathway [113]. In a mouse model with HCC induced with Hepa1-6 cells, RSV inhibited tumor growth by enhancing the production of anti-inflammatory cytokines and reducing the number of the immunosuppressive CD8^+^CD122^+^ Treg cells. Additionally, there was a decrease in the M2 macrophages [114]. HCC cells are resistant to the apoptotic and growth inhibition capacity of IFN-α. Yang et al. [115] have found that RSV increases the SMMC7721 liver cancer cells’ sensitivity to IFN-α through the SIRT/STAT1 pathway, which in turn promotes apoptosis and inhibits cells’ proliferation. Administration of RSV to hepatic cancer cells inhibited their growth and migration by promoting the activity of the autophagosomal proteins LC3II/I ratio, beclin 1, and the tumor suppressor protein P53 [116]. Tong et al. [117] discovered that RSV may impede Huh7 HCC cells’ development and migration by preventing the Rab27a protein-exosome creation, which plays a significant role in tumor development. A recent research showed that by activating the SIRT1/Nrf2 pathway in a HCC cell line, RSV may upregulate the activity of human telomerase inhibitor-telomerase reverse transcriptase (HepG2), thus reducing cancer cell survival [118]. Concurrent treatment of the HePG2 hepatocellular carcinoma cell line with RSV and berberine had a stronger inhibitory effect on cell viability, development, and apoptosis [119]. The fact that Hep-3B-p53 mutants were more susceptible to this combination suggests that p53 plays a crucial role in the growth of HCC [120]. HCC is resistant to most of the anticancer drugs and actually, the one that cancer cells may respond to is sorafenib. Research conducted *in vitro* using HepG2 and Hu7 hepatic cancer cell lines, as well as animals with HepG2-induced tumors showed that the combined treatment of sorafenib and RSV had a stronger anticancer effect than that of each component alone. A greater number of cells in the S phase mitotic arrest and apoptosis were indicative of this effect. The AMPK-activated protein kinase (PKA/AMPK/eEF2K) pathway was proposed as the underlying mechanism of this combination [121]. Table 1 illustrates the vast range of RSV dosages used *in vitro* and animal models and their primary impact on cells derived from digestive tract cancers. Further research presents a significant scientific challenge, as does figuring out an effective, non-toxic dose for humans.

**Table 1:** Effects of resveratrol on digestive system cancers

Type of cancer	Carcinoma cells	Animals	RSV dosage	RSV effect	Ref.
Esophageal	EC-9706		10 mM/L for 24–96 h	Promotes apoptosis, decreases Bcl-2 level	[82]
Esophageal	OE33, OE19, FLO-1		1–100 µM for 72 h	Decreases viability and Bcl-2, increases ROS	[83]
Gastric	SGC7901		0–100 µM	Inhibits IL-6 and Raf/MAPK pathway	[85]
Gastric	MGC-803		1–100 µM for 24, 48, 72 h	Downregulates Wnt pathway, cyclin D1, β-catenin	[86]
Gastric	SGC7901		25–50 mM	Induces apoptosis, cleaves PARP, increases Bax/Bcl-2	[87]
Gastric	SGC7901		200 µM	Inhibits viability, upregulates BAX, downregulates Bcl-2 and NF-kB	[88]
Gastric	AGS, MKN45		0–200 µM for 24 h	Inhibits cell invasion, NF-kB, and heparanase activity	[90]
Gastric	MGC803, SGC7901, GES-1		0–200 µM for 24 h	Inhibits cell proliferation and invasion by downregulating miR--155-5p expression	[91]
Gastric	SGC7901		5, 10, 20 µM	Inhibits TGF-β1 induced EMT	[92]
Colorectal	HT-29, RKO		10, 25, 60 µM	Alters the cross-talk between immune and cancer cells	[23]
Colorectal	HCT116, HT29		100 µM	Inhibits cell growth, activates Raf-1 (RKP) protein	[98]
Colorectal	HCT116, HT29, SW480		5, 10, 100 mM	Regulates the Wnt β-catenin pathway	[99]
Colorectal	HCT116, HT29		100 µM	Inhibits ENaCs by AMPK activation	[101]
Colorectal	HCT116		1, 2, 5, 10 µM	Activates SIRT and inhibits cell survival and migration	[104]
Pancreatic		Mice	50 mg/kg	Inhibits tumorigenesis by impeding NF-kB activation	[105]
Pancreatic	EPP85-181P. EPP85-181RNOV, AsPC-1		25, 50, 100 µM	Inhibits proliferation, alters the Bcl2 proteins	[107]
Pancreatic	Panc-1, MiaPaCa-2, BxPC-3, CF PAC-1, and SW1990	Mice	50–100 mM	Inhibits invasion and migration by suppressing NAF-1	[108]
Pancreatic	MIA-Pa-Ca-3		5–10 µM	Combined with phytochemicals activates Nrf2	[109]
Pancreatic	AsPC-1, BxPC-3, Capan2		20–50 µM	RSV derivative inhibits proliferation by DNA damage	[110]
Hepatocellular	Hepa1-6, H22	Mice	50 mg/kg	Decreases tumor growth, Inhibits CD8^+^CD12^+^ Tregs	[114]
Hepatocellular	SMMC7721		10, 20, 30 µM	Reduces apoptosis, activates SIRT1/STAT1	[115]
Hepatocellular	MHCC97-H		10–200 µM for 24 or 48 h	Autophagy, increases beclin1 and LC3 II/I ratio	[116]
Hepatocellular	Huh7		80 µM	Suppresses proliferation and migration and exosome secretion via Rab27A	[117]
Hepatocellular	HepG2		0, 10, 20, and 40 μM for 72 h	Upregulates hTERT induces SIRT1/Nerf2 pathway	[118]
Hepatocellular	Hep-3B, Hep-G2		0–10 µg/mL	Reduces viability by increasing p53 apoptosis	[120]
Hepatocellular	HepG2, Huh HepG2 HepG27	Mice	0–100 µM	Mitotic arrest and apoptosis via PKA/AMP/eEFF2K pathway	[121]

## Conclusions

Inhibition of cancer cells’ proliferation and migration and achieving their death should be essential goals in cancer treatment strategy. Unfortunately, cancer remains a challenging disease to treat even with major advancements in immune- and chemotherapy. The review presented herein provides significant insights into the mechanisms by which RSV demonstrates its anti-tumor properties. Notably, elucidating certain constraints of RSV as an anticancer agent, like inadequate pharmacokinetics, and lack of appropriate dose in humans, ought to showcase upcoming viewpoints [122]. Clarifying RSV’s benefits for cancer treatment will require addressing the low bioavailability issue and finding new methods to deliver RSV to the cells. A combination of anticancer drugs with plant polyphenols such as RSV, expressing both immune and anti-proliferative activities could be a feasible adjuvant in the anti-cancer pharmacopeia. It is necessary to move from *in vitro* experiments to further animal and clinical studies to achieve this goal.

## Data Availability

Not applicable.
